# Tumor endothelial cell-derived extracellular vesicles contribute to tumor microenvironment remodeling

**DOI:** 10.1186/s12964-022-00904-5

**Published:** 2022-06-25

**Authors:** Jian Gao, Xiaodong Zhang, Lei Jiang, Yan Li, Qianqian Zheng

**Affiliations:** 1 Department of Pathophysiology, College of Basic Medical Science, China Medical University, Shenyang, 110122 China; 2 Science Experiment Center of China Medical University, Shenyang, 110122 China; 3 Department of General Surgery, Beijing Friendship Hospital, Capital Medical University, Beijing, 100000 China; 4 National Clinical Research Center for Digestive Diseases, Beijing, 100000 China; 5 Department of General Surgery, Affiliated Hospital of Jinzhou Medical University, Jinzhou, 121000 China; 6 Department of Radiotherapy, The First Affiliated Hospital of Jinzhou Medical University, Jinzhou, 121000 China

**Keywords:** Extracellular vesicles, Tumor microenvironment, Angiogenesis, Remodeling, Targeted therapy

## Abstract

Cancer progression involves several biological steps where angiogenesis is a key tumorigenic phenomenon. Extracellular vesicles (EVs) derived from tumor cells and other cells in the tumor microenvironment (TME) help modulate and maintain favorable microenvironments for tumors. Endothelial cells (ECs) activated by cancer-derived EVs have important roles in tumor angiogenesis. Interestingly, EVs from ECs activate tumor cells, i.e. extracellular matrix (ECM) remodeling and provide more supplements for tumor cells. Thus, EV communications between cancer cells and ECs may be effective therapeutic targets for controlling cancer progression. In this review, we describe the current knowledge on EVs derived from ECs and we examine how these EVs affect TME remodeling.

**Video abstract**

**Supplementary Information:**

The online version contains supplementary material available at 10.1186/s12964-022-00904-5.

## Background

Cancer is a complex multifactorial disease where normal cells acquire multiple traits to facilitate prolonged cell survival [1]. Tumor occurrence and development is inseparable from the tumor microenvironment (TME) which is a highly heterogeneous ecosystem incorporating: 1) immunocytes (T/B lymphocytes, tumor-associated macrophages, dendritic cells (DCs), natural killer cells (NKs), myeloid-derived suppressor cells, and neutrophils); 2) stromal cells (cancer-associated fibroblasts (CAF), pericytes, and mesenchymal stromal cells); 3) extracellular matrix (ECM) and other secreted molecules (extracellular vesicles (EVs), growth factors, cytokines, and chemokines); and 4) blood and lymph vessel networks [2]. Such cell-to-cell interactions can lead to TME remodeling and may exert a significant impact on cancer progression and metastasis, drug resistance, and immunosuppression.

Recently EVs, also known as exosomes (Exs), have been recognized as crucial signaling mediators in TME regulation. EVs are double-membrane vesicle-like bodies which are shed from cell membranes or secreted by cells, with diameters ranging from 40 to 1000 nm. EVs are mainly composed of microvesicles (MVs) and Exs [3]. To explore this topic more comprehensively, the terms “EVs” and “Exs” are used interchangeably. EV-mediated communication networks between tumor and non-tumor cells appears to be involved from tumor growth to metastasis. For example, a gastric cancer study reported that the EV cargoes inhibin subunit βA and thrombospondin 2 were secreted by CAF-derived EVs to the TME to promote cancer [4]. Glioblastoma-derived EVs dramatically promoted neural progenitor cell proliferation and migration via the PI3K/Akt pathway [5]. A recent study also reported that EV transmitted long-non coding (lnc)ARSR which promoted sunitinib resistance in renal cancer [6]. EVs released by tumor cells could also remodel the microenvironment by activating CAF [7] and promoting immune escape [8] and angiogenesis [9]. Among the multiple stromal cell types in the TME, endothelial cells (ECs) are an enriched source of circulating EVs as they transfer information to adjacent cell types [10].

Several studies have now demonstrated that stromal cell-derived EVs promote tumorigenic phenotypes and that tumor-derived EVs modify the host stroma. However, the effects of EVs released from ECs on TEM are rarely studied. In this review, the role of EC-derived EVs is highlighted to examine their contribution to tumor cells and immune cells.

## Loading and releasing EVs

Previous studies have confirmed that endosome sorting complexes required for transport (ESCRT) are widely accepted regulatory mechanisms for EV processing, formation, and release. ESCRTs are composed of ESCRT-0, ESCRT-I, ESCRT-II, ESCRT-III, and vacuolar protein sorting-associated protein 4 (VPS4) [11]. ESCRT-0 initiates pre-ubiquitinated protein sorting and forms intraluminal vesicles (ILVs). Under the action of ESCRT-I/II, ILVs undergo membrane fusion to form multi-vesicular bodies (MVBs). Inside the cell, MVB parts are degraded by lysosomes, while the remaining components move toward cell membranes and fuse with them. At this time, MVBs are still connected to the membrane surface and ESCRT-III forms a spiral structure during this process. This structure shrink the MVB neck and the cell membrane. At the same time, the VPS4 ATPase directly or indirectly hydrolyzes the MVB through hydrolysis, after which MVBs are released extracellularly. EVs are also formed via an ESCRT-independent mechanism; Pmel17 regulates ILV production and affects EV formation via its luminal domain [12]. Also, the four-transmembrane CD63 protein mediates melanosome invagination in an ESCRT-independent manner [13]. The PLP protein is transferred from lipid-rich endosomal membranes to ILVs via an ESCRT-independent manner [14]. In addition to ESCRT-mediated pathways, Wei et al*.* identified phosphorylates RAB31 drives EGFR entry into multivesicular endosomes (MVE) to form ILVs and Exs, which is dependent of flotillin proteins in lipid raft microdomains instead of ESCRT [15].

**Fig. 1:**
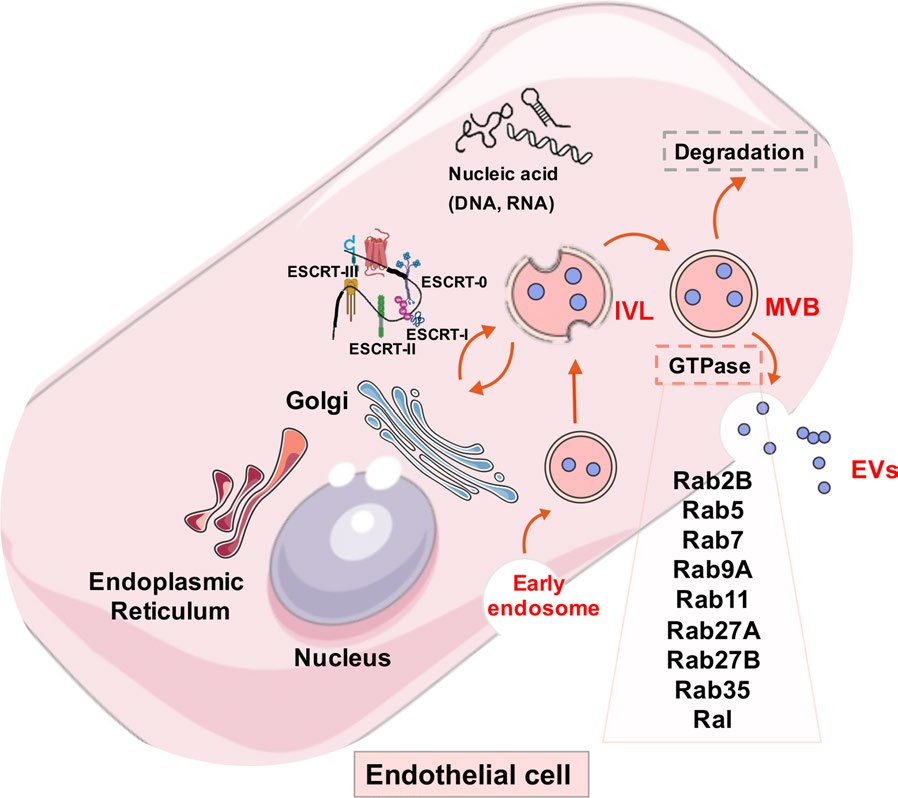
Schematic overview of extracellular vesicle uptake and release

EV-release from inside the cell to the outside is synergistically coordinated via several steps which mainly act on MVB and cell membrane separation processes. Studies have confirmed the most important factors mediating EV release are GTPases, including Rab and RAL GTPases. Up to now, nine GTPases have been implicated in EV release, including Rab2B, Rab5, Rab7, Rab9A, Rab11, Rab27A, Rab27B, Rab35, and RAL [16]. Rabs have vital regulatory roles transporting MVBs to subcellular locations and fusing with cell membranes [17]. MVBs can be directly or indirectly bound to actin and microtubule scaffolds to facilitate intracellular targeted transport. Interestingly, Rab11 and its family assist MVB transport via actin and dynein [18]. The process from MVBs to EVs requires not only motor protein dynamics, but also MVB separation from cell membranes which is particularly important. Although the specific mechanisms whereby Rab participates in MVB dissociation at the cell membrane have not been confirmed, it is hypothesized Rab initiates the direct or indirect assembly of soluble N-ethylmaleimide-sensitive factor attachment protein receptor (SNARE) complexes (Fig. 1).

The SNARE complex has vital roles in MVB fusion with cell membranes, especially when promoting MVB release. The SNARE complex consists of two components, v- and t-SNAREs. The v-SNARE is located on the vesicle while the t-SNARE is located on the presynaptic membrane. Both v- and t-SNAREs pair up and form a complex [19]; during this formation, the released energy draws MVBs closer to presynaptic membranes and promotes MVB membrane fusion. A recent study in human leukemic K562 cells confirmed that VAMP7 (a v-SNARE protein) was involved in MVB plasma membrane fusion and exosomal release [20]. In mammals, SYX-5 (a t-SNARE proteins) was also involved in MVB fusion and promoted EV release [21]. In tumor cells, the key glycolysis enzyme PKM2 stabilized SNAREs via SNAP-23 (a t-SNARE protein) phosphorylation to promote external EV release [22]. These studies confirmed that mediating EV release was a complex process involving multiple steps and factors. While the regulatory mechanism is not the same in different cells, the main processes often involve GTPases and SNARE complexes. These observations raise an important question in EV research: do different sorting mechanisms determine the loading of specific molecules into EVs? By interfering with this sorting mechanism, it is possible to influence content loading in EVs to alter intercellular substance exchange in the TME.

## EV uptake

EVs released into the extracellular space are not only re-absorbed and used by EV-derived origin cells, but are also taken up by other cells in the microenvironment. Cells take up EVs mainly via cell membrane fusion, endocytosis, and binding with specific surface receptors. Additionally, when carrying functional molecules, EVs transfer information between cells and mediate many physiological and pathological processes. Tian et al*.* reported that rat pheochromocytoma PC12 cell-derived Exs entered and delivered microRNAs into bone marrow-derived mesenchymal stromal cells and down-regulated the expression of transforming growth factor β receptor II and tropomyosin-1 [23]. In non-neoplastic diseases, ECs transduced with HDAdXMoAntimiR33a5p released Exs that transferred anti-miR-33a-5p to other intimal cell types, thereby upregulating cholesterol efflux from these cells. [24]. At present, the decisive factors determining EV uptake remain unclear. Edgar et al*.* reported that the protein tether in mediating the attachment to the cell surface and signal transmission of EVs [25]. Christianson et al*.* confirmed that on the surface of receptor cells, the heparin sulfate proteoglycan (HSPG) acted as an EV receptor rather than just an attachment site on the EV surface [26]. In mammals, differences in integrin family expression often affect EV uptake by receptor cells [27]. Importantly, some mechanisms can inhibit uptake; CD47 on EV surfaces protects EVs from engulfment by recipient cells and improves EV stability in the microenvironment [28].

Tumor-derived EVs taken up by ECs often facilitate angiogenesis signaling and stimulate blood vessel formation [29]. In ECs, EV take-up is interceded via the collaboration of EV surface proteins, e.g., tetraspanins with the membrane receptors of beneficiary cells [30]. In the TME, cell surface Tspan8-CD49d complexes from EVs derived from tumors are incorporated by vascular rodent ECs to improve EC proliferation, migration, and activation [31]. EVs bearing Tspan8-4 complexes bind with intercellular adhesion molecule-1 (ICAM-1) and then were compassed by rat ECs [32]. In the absence of content delivery, EVs send messages to beneficial cells via surface contacts, e.g., EVs containing major histocompatibility complex (MHC)-peptide complexes can activate T cells via surface receptors [33]. Within 24 h, ECs internalize EVs produced by cancer cells using an internalization pathway. This was previously confirmed in studies where ECs easily captured PKH26-dyed EVs during the first 4 h [34]. After internalization, EVs were immediately directed to the perinuclear zone; they moved to the cell periphery and entered advanced pseudopods when tubules were formed in vitro. After complete remodeling, adjacent ECs transported EVs to other ECs and cells in the TME via nanoparticle structures [35].

## EC-derived EV characterization, separation, and roles in tumor progression

**Fig. 2:**
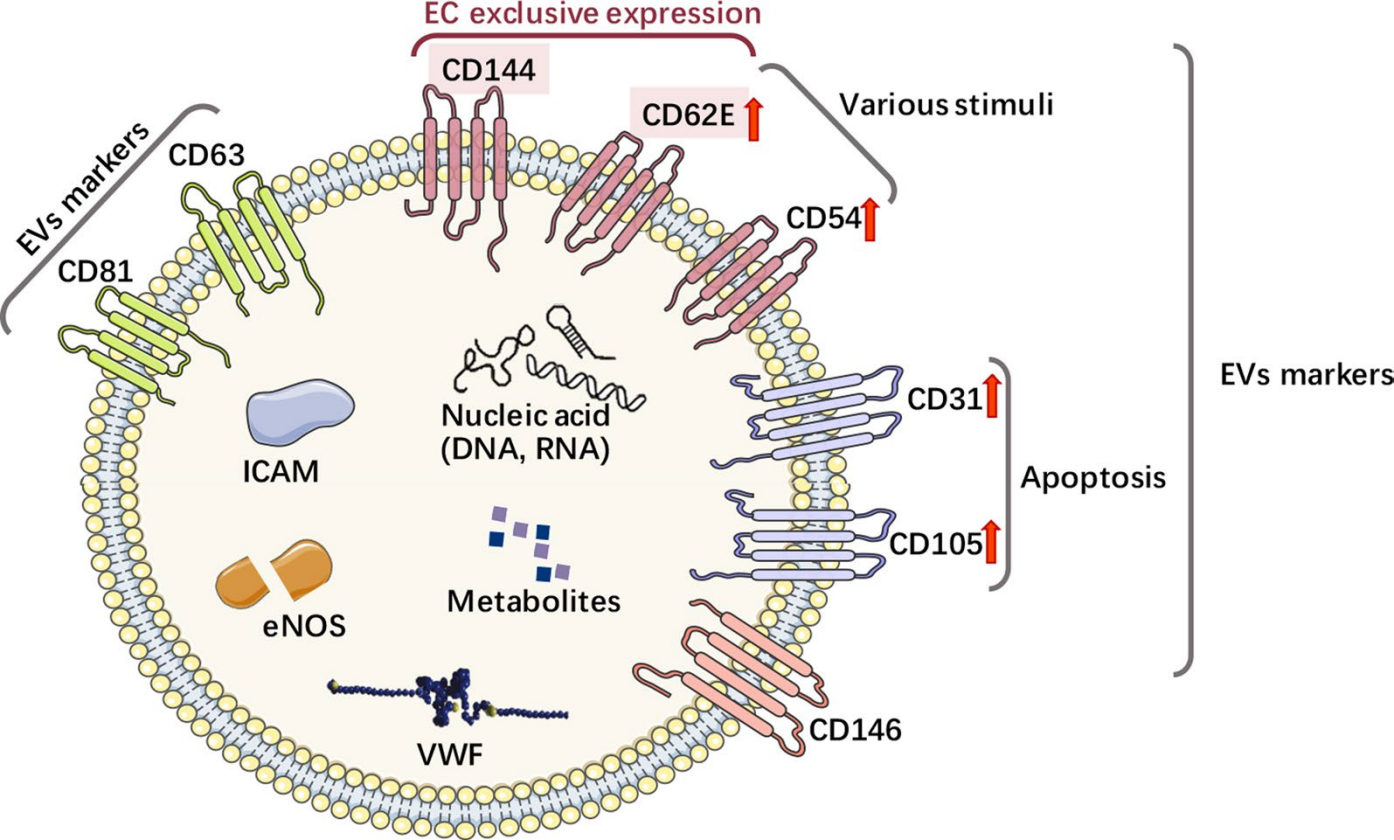
Specific markers of endothelial cells-derived EVs

EVs derived from ECs are implicated in several physiological and pathological conditions [36–38]. The particulate secretome of endothelia mirrors their molecular diversity and possibly supports EC plasticity and adaptation within or between different vascular beds while also significantly impacting circulating cells, including immune cells in blood or lymph [39]. The markers CD63 and CD81 are specifically expressed in EVs. In addition, several other EV-specific markers, i.e. CD31, CD54, CD62E, CD105, CD144, CD146, and von Willebrand factor also occur on EVs [32, 33]. Moreover, EC-derived EVs also deliver proteins such as ICAMs, vascular endothelial (VE)-cadherin, E-selectin, platelet EC adhesion molecule-1, endoglin, and endothelial nitric oxide synthase [40–42]. While the exosomal endothelial markers CD54 and CD62E are upregulated by various stimuli, CD31 and CD105 are specifically increased by apoptosis [43]. CD62E and CD144 are exclusively expressed by ECs, while the aforementioned molecules are not (Fig. 2). To improve specificity and sensitivity, combined multicolor antibodies (CD31^+^/CD41^−^, CD31^+^/CD42b^−^, and CD105^+^/CD45^−^) and monochrome composite markers (CD144^+^ CD105^+^ and CD146^+^ CD105^+^) are used to isolate EC-derived EVs. Because ECs express CD62E and CD144 [43], EC-delivered EVs can be specifically separated, identified, and quantified from plasma and various tissues by immunoaffinity (CD144/CD62E antibodies) or nanoscale fluorescence-activated cell sorting, which is a highly promising and rapidly advanced method for EV separation and characterization [44].

**Fig. 3:**
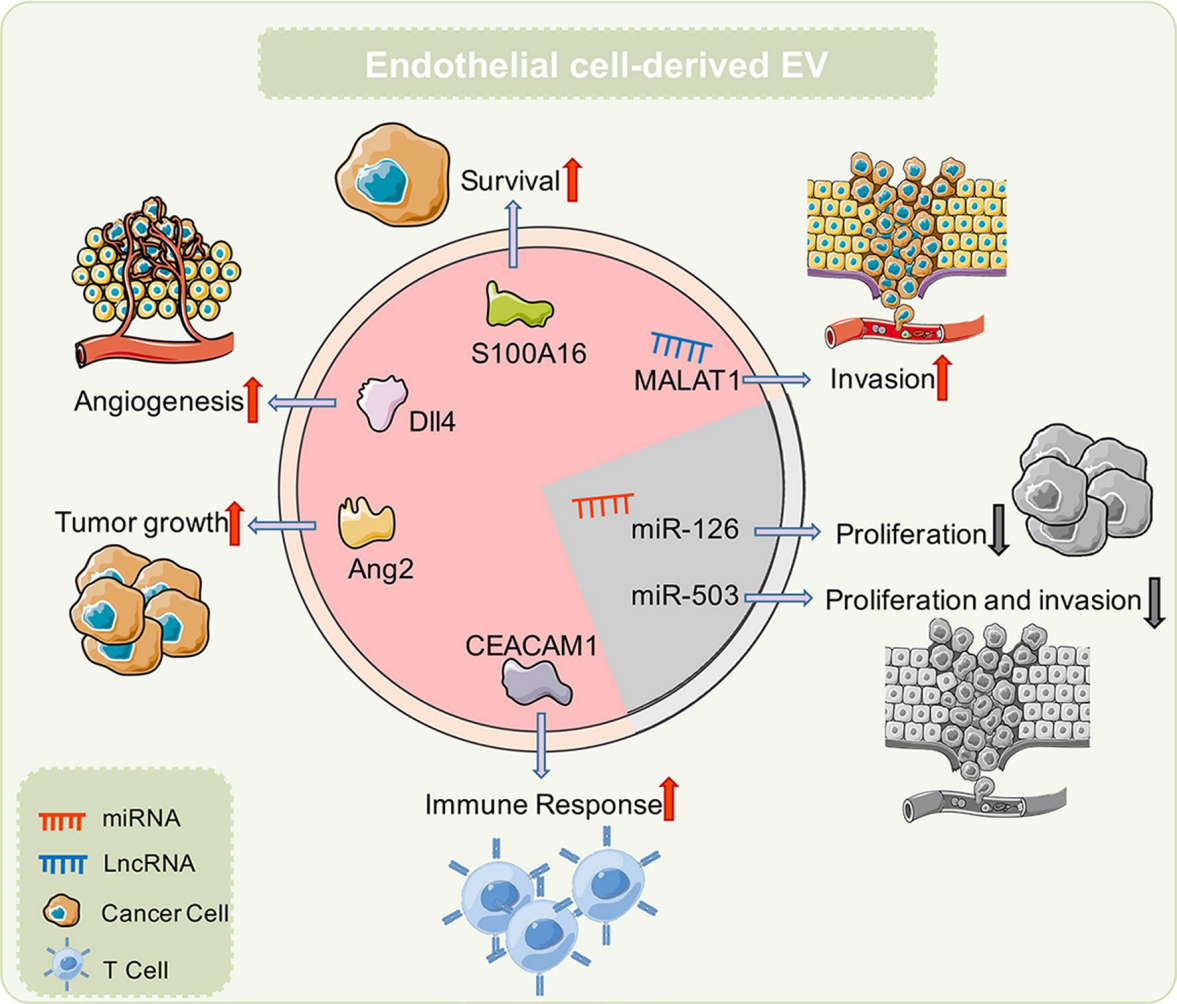
Schematic molecular components of endothelial cells-derived EVs

EV cargoes are closely related to donor cells, and the substances in EVs released by different cells often have significant differences [45]. Current research has confirmed that EVs released by ECs contain high levels of lncRNAs and protein components. These contents exert different functions during cancer; some inhibit cancer progression, including miR-503 [46] and miR-126 [47], while others promote cancer progression, including MALAT1[48], S100A16 [49], delta-like protein 4 [50], angiopoietin-2 (Ang2) [51], and carcinoembryonic antigen-related cell adhesion molecule-1 (CEACAM1) [52]. For non-neoplastic diseases, EV contents also exert important regulatory roles as they mediate EC function, with miR-125a [53], miR-210 [54], miR-375 [55], miR-214 [56], lysyl oxidase like-2 (LOXL2) [57], and HSP70 [58] having significant roles in this process. In heart disease, miR-10b-5p [59], miR-146a [60], and miR-19a [61] are important molecules, while for vascular smooth muscle cell regulation, miR-143 [62] and versican [63] are highly significant. In atherosclerosis, miR-505 [64] and miR-155 [65] also have similarly important functions. In chronic obstructive pulmonary disease, R-191 predicts the potential function of Exs as paracrine effectors [53]. TGF-β1 could ameliorate renal structure and function [66]. Cytomegalovirus could stimulate allogeneic CD4^+^ memory T cells [67]. The effects of EV release on tumor cells is mainly facilitated via these cargoes. e.g., miR-503 in EVs regulate the proliferation and invasion of triple-negative breast cancer cells [46]. Also, stressed human umbilical vein endothelial cells (HUVECs) release EVs containing miR-126, which significantly inhibits tumor cell growth [47]. In addition to releasing “tumor-suppressing” EVs, ECs also release “tumor-promoting” EVs. The EVs released from human brain microvascular ECs promote S100A16 expression in lung cancer cells, increase lung cancer cell and anti-apoptotic activity, thereby promoting lung cancer development. ECs also release EVs carrying Ang2 which promotes tumor progression [51]. Under stress conditions, CEACAM1 in EVs inhibits T cell activation, which may be an important factor promoting tumor progression. Our previous studies showed that YAP1 inhibition in HUVECs was associated with EV release and increased hepatocyte carcinoma (HCC) invasion and metastasis (Fig. 3). Our results suggested that during tumor treatment, EVs were a possible reason for treatment failure [48] (Table 1).

**Table 1:** The role of EC-derived EVs in tumor and non-tumor diseases

		Parent cell	Biological function	Reference
*EVs on tumor cells*				
NcRNA	miR-503	HUVECs	Inhibits cancer proliferation and invasion	[46]
	miR-126	HUVECs	Inhibits the growth of tumor cells	[47]
	MALAT1	HUVEC	Promotes invasion in HCC	[48]
Protein	S100A16	HBMECs	Promotes SCLC survival in brain	[49]
	Dll4	HMVECs	Promotes angiogenesis	[50]
	Ang2	Primary mouse lung endothelial cells	Promotes tumor growth	[51]
	CEACAM1	human endothelial cell line AS-M.5	Modulate immune response, tumor progression, metastasis and angiogenesis	[52]
*EVs on non-neoplastic diseases*				
NcRNA	miR-10b-5p	HUVEC	Reduced inflammation in cardiovascular disease	[59]
	miR-125a	Human lung microvascular	Modulate the phenotype of distant endothelial cells of large systemic	[53]
	miR-143	HUVEC	Control smooth muscle cell phenotype	[62]
	miR-146a	HUVEC	Decrease in metabolic activity in peripartum cardiomyopathy	[60]
	miR-191	Human lung microvascular	Predicting potential function of exosomes as paracrine effectors in COPD	[53]
	miR-210	Endothelial progenitor cells	Protective effects on ECs against H/R injury	[54]
	miR-375	Endothelial progenitor cell	Rescued the cell protection activity	[55]
	miR-214	HMEC-1	Blood vessel formation	[56]
	miR-505	HUVEC	Inducing Oxidative stress and inflammation in atherosclerosis	[64]
	miR-155	HUVEC	Modulate the macrophage phenotype in atherosclerosis	[65]
	miR-19a	HUVEC	Improved vascularization and cardiac function, decreased myocardial fibrosis	[61]
Protein	TGF-β1	Glomerular endothelial cells	Ameliorate renal structure and function	[66]
	Versican	HUVEC	Regulate vascular smooth muscle cells calcification/senescence in high glucose condition	[63]
	LOXL2	Human microvascular endothelial cells	Altered abundances after exposure of their producing cells to cellular stress	[57]
	Cytomegalovirus	HUVEC	Stimulate allogeneic CD4 + Memory T Cells	[67]
	HSP70	Rat aortic endothelial cells	Induction of monocyte activation and endothelial cell adhesion	[58]

## The role of EVs toward immune cells

EVs trigger and regulate immune responses, especially in cancer [68]. Several studies have reported that various molecules (adhesions, heat shock proteins, molecules involved in membrane trafficking and ESCRTs), immune response molecules (e.g., MHC class I and II proteins), immune receptors, ligands (FasL, TRAIL, PD-L1, NKG2D ligands), and co-stimulatory molecules are distinct cargoes in some specialized EVs. Many immunocytes, such as DCs [69], NKs, and T cells [70] are activated by ECs harboring non-classical or HLA-E, MICA, and other NKG2D ligands. In cardiovascular disease, including vascular inflammation and atherosclerotic plaque formation, EVs mediate cross-talk between recipient cells and ECs, and reprogram them toward a pro- or anti-inflammatory stance. The pro-inflammatory molecules with chemotactic mediators, including ICAM-1, CCL-2, IL-6, IL-8, CXCL-10, CCL-5, and TNF-α were released by EVs and taken up by monocytes and HUVECs [71]. In anti-inflammatory aspect displays, ECs released EVs containing miRNA-222 to reduce endothelial ICAM-1 expression both in vitro and in vivo [72]. In addition, miR-10a was transferred to monocytes from EC-EVs and could repress inflammatory signaling through the targeting of several components of the NF-κB pathway, including IRAK4 [73]. Lipopolysaccharide induces the neutrophil secretion of EVs containing miR-122-5p which functions in oxidative stress, apoptosis, and increased brain microvascular EC permeability [74]. EVs released by apoptotic ECs are reported as having unique transcriptomic characteristics and contain non-coding RNA (ncRNA) sequences (mitochondrial transfer RNA, U1 small nuclear RNA, and pathogen-like endogenous retro-elements) with immunostimulatory potential. These EVs from apoptotic ECs may be recognized by RIGI-like receptors and toll like receptors (TLRs) (TLR3, TLR7, and TLR8) and possibly initiate innate immune responses [75].

The circuitous connection between certain EVs and tumors warrants further investigation. Specific ncRNAs have been identified as cargoes in specialized EVs released by ECs; interestingly, the same ncRNAs regulate tumor progression in other studies. We have no proof of a relationship between EC-EV-ncRNA-tumor, yet this gives us a sensible estimate concerning the connection among these elements. Zhao et al*.* found that miR-503 downregulated immune function in esophagus carcinoma [76], while Bovy et al*.* confirmed miR-503 was contained in EC EVs during breast cancer neoadjuvant chemotherapy [46]. LncRNAs also have important regulatory roles in tumor immunity. We previously demonstrated that ECs release EVs which carried MALAT1 [48], while Hou et al*.* reported that MALAT1 promoted immunosuppressive properties in HCC cells [77]. Primary mouse lung ECs may release EVs containing Ang2, while coincidentally, Schmittnaegel et al*.* verified that ANG-2 inhibition elicited antitumor immunity which was enhanced by PD-1 checkpoint blockade [78]. In addition to ncRNAs, proteins from EVs released by ECs also have important functions in tumor immunity. S100A6 overexpression possibly impaired the infiltration and cytolytic activity of CD8^+^ T cells via the focal adhesion-Ras-stimulating signaling pathway in pancreatic cancer [79]. Brain microvascular EC-EVs contained S100A16 which is an immune-related prognostic biomarker and therapeutic target for low-grade glioma [80]. These results suggest EVs released from ECs may be important factors regulating tumor immunity. Anti-tumor immunotherapy targeting EC-EVs may an important anti-tumor therapy mechanism in the future.

## EC derived EV based therapeutic strategies

EVs containing various surface adhesion proteins have several advantages as delivery vectors for cancer gene therapy. As cancers are often associated with aberrant EV formation and release, the inhibition of this pathological process could be an emerging approach for cancer treatment.

### EVs as novel biocarriers for drug delivery

EC-derived EVs are advantageous for drug targeted delivery as they carry biological substances to recipient cells. Using in vitro and ex vivo techniques, including freeze-thaw cycles, incubation, saponin infiltration, sonication, and extrusion procedures, several therapeutic drugs have been loaded into EVs [81]. Thus, EVs are stable, and drug structure and activity remains unchanged after EV loading, even when stored at freezing temperatures. Recent studies suggested that EV delivery and effective cellular uptake could be improved by altering the EV surface with an Arg-Gly-Asp-D-Tyr-Lys peptide [82] or promoting efficient cytosolic release using enhancing cationic lipids [83]. Unfortunately, few studies have reported EC-derived EVs for delivering drugs in experimental or clinical studies, while other cell studies reported that EVs carrying anticancer drugs are promising new therapeutic strategies in animal experiments as a promising approach for cancers [84]. In addition to undergoing a phase I clinical trial (NCT01294072), these therapeutic strategies are being used for EC/EV drug delivery for cardiovascular diseases.

### EVs carry small interfering RNAs (siRNAs)

EVs carry RNAs, which facilitate communications between cells. Critically, siRNA delivery to ECs and other cells could be an important gene therapy strategy. Because protein expression is suppressed by siRNAs via specific posttranscriptional sequences, siRNAs offer a unique opportunity to treat multiple ECs related to cancer. Recently, across different fields, several siRNA vectors have been used, including viruses, synthetic polymers, and lipid-based carriers. When compared with other RNA vectors, EVs when used as nanoparticles to transfer siRNAs, are more advantageous in retaining bioavailability, delivery efficiency, and compatibility [85]. Moreover, siRNA-containing EVs cross the endothelial barrier and guide siRNAs to target specific tissues and cells [85]. Also, as these molecules easily pass the blood–brain barrier, EVs are extensively used as small RNA carriers to treat brain tumors [86]. A recent study showed that EVs containing siRNA knocked down target gene expression by 50%–90% in different cancer cells [87]. However, few investigations have provided evidence of specific ECs releasing EVs to deliver siRNA. Yang et al*.* demonstrated that siRNA-VEGF delivery in EC-EVs suppressed VEGF transcription and translation in co-cultured GBM-astrocytoma cells, and showed that siRNA delivery silenced target genes and could be used as a potential therapeutic strategy for cancer [88].

### Reducing the abnormal release of EVs

In terms of abnormal EV-release related to cancer, several studies emphasized the biological ways of EVs to verify the key way to suppress the exocrine of EVs. nSMase took part in lipid raft and worked as a ubiquitous enzyme to improve EV formation [89]. These results suggested EV formation and release were blocked by siRNAs against nSMase2. Menck et al*.* reported that nSMase-2 inhibition by GW4869 or RNA interference decreased Exs secretion but increased MV secretion from plasma membranes both in vitro and in vivo [90]. Furthermore, GW4869 prevented abnormal EV release and improved abnormal vascular remodeling [91]. Rab proteins are key regulators of EV secretion, e.g., Rab27 participates in EV exocytosis. Also, EV release is reduced by RAB27 inhibition [92]. In colorectal cancer cells, RAB27 knockdown inhibited EV release and the exosomal-related proliferation and migration of ECs [93].

## Conclusions and perspectives

Angiogenesis is an essential step in tumorigenesis and development. Tumor cell metastasis to distant organs and the rapid proliferation of focal tumor cells are inseparable from new blood vessel formation. EVs are important mediators during material exchange processes between tumor cells and ECs. EVs released by tumor cells carry proteins and ncRNAs to promote new tumor blood vessel formation. ECs in the TME also release EVs to promote tumor progression. Currently, the function and molecular mechanisms underpinning the EC release of EVs are unclear and require further study. However, EV sources, their loading, and related processes are affected by their cell origin, changes in the TME, and target cells. Therefore, targeting these EV characteristics could provide an intervention for regulating tumor angiogenesis. In particular, EVs are produced in the body and effectively avoid immune surveillance, therefore, they have a great potential as a targeted therapy for drug delivery in clinical settings. Based on these observations, cross-talk between ECs and other cells can be mediated by EVs and contribute to TME remodeling. EC-derived EVs are expected to become important targets for tumor treatments in the future.

## Abbreviations

EVs: Extracellular vesicles
TME: Tumor environment
TAM: Tumor-associated macrophages
DC: Dendritic cells
NK: Natural killer cells
MDSC: Myeloid-derived suppressor cells
CAFs: Cancer-associated fibroblasts
ECM: Extracellular matrix
MVs: Microvesicles
Exs: Exosomes
MVE: Multivesicular endosomes
ECs: Endothelial cells
HCC: Hepatocyte carcinoma
ESCRT: Endosome sorting complexes required for transport
MVB: Multi-vesicular body
VPS4: Vacuolar protein sorting-associated protein 4
ILV: Intraluminal vesicles
MVB: Multivesicular bodies
SNARE: Soluble N-ethylmaleimide-sensitive factor attachment protein receptor
ANGPT1: Angiopoietin-1
BMSCs: Bone marrow-derived mesenchymal stromal cells
TGFβRII: Transforming growth factor β receptor II
TPM1: Tropomyosin-1
HSPG: Heparin sulfate proteoglycan
CA9: Carbonic anhydrase 9
miRNA: MicroRNA
lncRNA: Long-noncoding RNA
VWF: Von Willebrand factor
ICAM: Intercellular cell adhesion molecule
MHC: Major histocompatibility complex
PECAM-1: Platelet EC adhesion molecule-1
eNOS: Endothelial nitric oxide synthase
nanoFACS: Nanoscale fluorescence activated cell sorting
Dll4: Delta-like protein 4
LOXL2: Lysyl Oxidase Like- 2
Ang2: Angiopoietin-2
CEACAM1: Carcinoembryonic antigen-related cell adhesion molecule-1

## References

[1] Tamura K, Utsunomiya J, Iwama T, Furuyama J, Takagawa T, Takeda N, Fukuda Y, Matsumoto T, Nishigami T, Kusuhara K (2004). Mechanism of carcinogenesis in familial tumors. Int J Clin Oncol.

[2] Oriuchi N, Sugawara S, Shiga T (2020). Positron emission tomography for response evaluation in microenvironment-targeted anti-cancer therapy. Biomedicines.

[3] Naseri M, Bozorgmehr M, Zöller M, Ranaei Pirmardan E, Madjd Z (2020). Tumor-derived exosomes: the next generation of promising cell-free vaccines in cancer immunotherapy. Oncoimmunology.

[4] Grunberg N, Pevsner-Fischer M, Goshen-Lago T, Diment J, Stein Y, Lavon H, Mayer S, Levi-Galibov O, Friedman G, Ofir-Birin Y (2021). Cancer-associated fibroblasts promote aggressive gastric cancer phenotypes via heat shock factor 1-mediated secretion of extracellular vVesicles. Cancer Res.

[5] Pan J, Sheng S, Ye L, Xu X, Ma Y, Feng X, Qiu L, Fan Z, Wang Y, Xia X, Zheng JC (2022). Extracellular vesicles derived from glioblastoma promote proliferation and migration of neural progenitor cells via PI3K-Akt pathway. Cell Commun Signal.

[6] Qu L, Ding J, Chen C, Wu ZJ, Liu B, Gao Y, Chen W, Liu F, Sun W, Li XF (2016). Exosome-transmitted lncARSR promotes sunitinib resistance in renal cancer by acting as a competing endogenous RNA. Cancer Cell.

[7] Ilkhani K, Bastami M, Delgir S, Safi A, Talebian S, Alivand MR (2020). The engaged role of tumor microenvironment in cancer metabolism: Focusing on cancer-associated fibroblast and exosome mediators. Anticancer Agents Med Chem.

[8] Schwich E, Hò GT, LeMaoult J, Bade-Döding C, Carosella ED, Horn PA, Rebmann V (2020). Soluble HLA-G and HLA-G bearing extracellular vesicles affect ILT-2 positive and ILT-2 negative CD8 T cells complementary. Front Immunol.

[9] Guo Z, Wang X, Yang Y, Chen W, Zhang K, Teng B, Huang C, Zhao Q, Qiu Z (2020). Hypoxic tumor-derived exosomal long noncoding RNA UCA1 promotes angiogenesis via miR-96-5p/AMOTL2 in pancreatic cancer. Mol Ther Nucleic Acids.

[10] Sharma A, Seow JJW, Dutertre CA, Pai R, Blériot C, Mishra A, Wong RMM, Singh GSN, Sudhagar S, Khalilnezhad S (2020). Onco-fetal reprogramming of endothelial cells drives immunosuppressive macrophages in hepatocellular carcinoma. Cell.

[11] Vietri M, Radulovic M, Stenmark H (2020). The many functions of ESCRTs. Nat Rev Mol Cell Biol.

[12] Babst M (2011). MVB vesicle formation: ESCRT-dependent, ESCRT-independent and everything in between. Curr Opin Cell Biol.

[13] van Niel G, Charrin S, Simoes S, Romao M, Rochin L, Saftig P, Marks MS, Rubinstein E, Raposo G (2011). The tetraspanin CD63 regulates ESCRT-independent and -dependent endosomal sorting during melanogenesis. Dev Cell.

[14] Mashouri L, Yousefi H, Aref AR, Ahadi AM, Molaei F, Alahari SK (2019). Exosomes: Composition, biogenesis, and mechanisms in cancer metastasis and drug resistance. Mol Cancer.

[15] Wei D, Zhan W, Gao Y, Huang L, Gong R, Wang W, Zhang R, Wu Y, Gao S, Kang T (2020). RAB31 marks and controls an ESCRT-independent exosome pathway. Cell Res.

[16] Song L, Tang S, Han X, Jiang Z, Dong L, Liu C, Liang X, Dong J, Qiu C, Wang Y, Du Y (2019). KIBRA controls exosome secretion via inhibiting the proteasomal degradation of Rab27a. Nat Commun.

[17] Peinado H, Alečković M, Lavotshkin S, Matei I, Costa-Silva B, Moreno-Bueno G, Hergueta-Redondo M, Williams C, García-Santos G, Ghajar C (2012). Melanoma exosomes educate bone marrow progenitor cells toward a pro-metastatic phenotype through MET. Nat Med.

[18] Ducharme NA, Ham AJ, Lapierre LA, Goldenring JR (2011). Rab11-FIP2 influences multiple components of the endosomal system in polarized MDCK cells. Cell Logist.

[19] Wang T, Li L, Hong W (2017). SNARE proteins in membrane trafficking. Traffic.

[20] Fader CM, Sánchez DG, Mestre MB, Colombo MI (2009). TI-VAMP/VAMP7 and VAMP3/cellubrevin: Two v-SNARE proteins involved in specific steps of the autophagy/multivesicular body pathways. Biochim Biophys Acta.

[21] Hyenne V, Apaydin A, Rodriguez D, Spiegelhalter C, Hoff-Yoessle S, Diem M, Tak S, Lefebvre O, Schwab Y, Goetz JG, Labouesse M (2015). RAL-1 controls multivesicular body biogenesis and exosome secretion. J Cell Biol.

[22] Williams KC, McNeilly RE, Coppolino MG (2014). SNAP23, Syntaxin4, and vesicle-associated membrane protein 7 (VAMP7) mediate trafficking of membrane type 1-matrix metalloproteinase (MT1-MMP) during invadopodium formation and tumor cell invasion. Mol Biol Cell.

[23] Tian T, Zhu YL, Zhou YY, Liang GF, Wang YY, Hu FH, Xiao ZD (2014). Exosome uptake through clathrin-mediated endocytosis and macropinocytosis and mediating miR-21 delivery. J Biol Chem.

[24] Stamatikos A, Knight E, Vojtech L, Bi L, Wacker B, Tang C, Dichek DA (2020). Exosome-mediated transfer of anti-miR-33a-5p from transduced endothelial cells enhances macrophage and vascular smooth muscle cell cholesterol efflux. Hum Gene Ther.

[25] Edgar JR, Manna PT, Nishimura S, Banting G, Robinson MS (2016). Tetherin is an exosomal tether. Elife.

[26] Christianson HC, Svensson KJ, van Kuppevelt TH, Li JP, Belting M (2013). Cancer cell exosomes depend on cell-surface heparan sulfate proteoglycans for their internalization and functional activity. Proc Natl Acad Sci U S A.

[27] Hoshino A, Costa-Silva B, Shen TL, Rodrigues G, Hashimoto A, Tesic Mark M, Molina H, Kohsaka S, Di Giannatale A, Ceder S (2015). Tumour exosome integrins determine organotropic metastasis. Nature.

[28] Kamerkar S, LeBleu VS, Sugimoto H, Yang S, Ruivo CF, Melo SA, Lee JJ, Kalluri R (2017). Exosomes facilitate therapeutic targeting of oncogenic KRAS in pancreatic cancer. Nature.

[29] Whiteside TL (2016). Tumor-derived exosomes and their role in cancer progression. Adv Clin Chem.

[30] Mulcahy LA, Pink RC, Carter DR (2014). Routes and mechanisms of extracellular vesicle uptake. J Extracell Vesicles.

[31] Nazarenko I, Rana S, Baumann A, McAlear J, Hellwig A, Trendelenburg M, Lochnit G, Preissner KT, Zoller M (2010). Cell surface tetraspanin Tspan8 contributes to molecular pathways of exosome-induced endothelial cell activation. Cancer Res.

[32] Rana S, Yue S, Stadel D, Zoller M (2012). Toward tailored exosomes: the exosomal tetraspanin web contributes to target cell selection. Int J Biochem Cell Biol.

[33] Tkach M, Kowal J, Zucchetti AE, Enserink L, Jouve M, Lankar D, Saitakis M, Martin-Jaular L, Thery C (2017). Qualitative differences in T-cell activation by dendritic cell-derived extracellular vesicle subtypes. EMBO J.

[34] Puzar Dominkus P, Stenovec M, Sitar S, Lasic E, Zorec R, Plemenitas A, Zagar E, Kreft M, Lenassi M (2018). PKH26 labeling of extracellular vesicles: Characterization and cellular internalization of contaminating PKH26 nanoparticles. Biochim Biophys Acta Biomembr.

[35] Gonda A, Kabagwira J, Senthil GN, Wall NR (2019). Internalization of exosomes through receptor-mediated endocytosis. Mol Cancer Res.

[36] Abid Hussein MN, Meesters EW, Osmanovic N, Romijn FP, Nieuwland R, Sturk A (2003). Antigenic characterization of endothelial cell-derived microparticles and their detection ex vivo. J Thromb Haemost.

[37] Combes V, Simon AC, Grau GE, Arnoux D, Camoin L, Sabatier F, Mutin M, Sanmarco M, Sampol J, Dignat-George F (1999). In vitro generation of endothelial microparticles and possible prothrombotic activity in patients with lupus anticoagulant. J Clin Invest.

[38] Martinez MC, Tesse A, Zobairi F, Andriantsitohaina R (2005). Shed membrane microparticles from circulating and vascular cells in regulating vascular function. Am J Physiol Heart Circ Physiol.

[39] Mathiesen A, Hamilton T, Carter N, Brown M, McPheat W, Dobrian A (2021). Endothelial extracellular vesicles: From keepers of health to messengers of disease. Int J Mol Sci.

[40] Dignat-George F, Boulanger CM (2011). The many faces of endothelial microparticles. Arterioscler Thromb Vasc Biol.

[41] Endemann DH, Schiffrin EL (2004). Endothelial dysfunction. J Am Soc Nephrol.

[42] Baruah J, Wary KK (2019). Exosomes in the regulation of vascular endothelial cell regeneration. Front Cell Dev Biol.

[43] Hromada C, Muhleder S, Grillari J, Redl H, Holnthoner W (2017). Endothelial extracellular vesicles-promises and challenges. Front Physiol.

[44] Morales-Kastresana A, Telford B, Musich TA, McKinnon K, Clayborne C, Braig Z, Rosner A, Demberg T, Watson DC, Karpova TS (2017). Labeling extracellular vesicles for nanoscale flow cytometry. Sci Rep.

[45] Charoenviriyakul C, Takahashi Y, Morishita M, Matsumoto A, Nishikawa M, Takakura Y (2017). Cell type-specific and common characteristics of exosomes derived from mouse cell lines: yield, physicochemical properties, and pharmacokinetics. Eur J Pharm Sci.

[46] Bovy N, Blomme B, Freres P, Dederen S, Nivelles O, Lion M, Carnet O, Martial JA, Noel A, Thiry M (2015). Endothelial exosomes contribute to the antitumor response during breast cancer neoadjuvant chemotherapy via microRNA transfer. Oncotarget.

[47] Wu X, Liu Z, Hu L, Gu W, Zhu L (2018). Exosomes derived from endothelial progenitor cells ameliorate acute lung injury by transferring miR-126. Exp Cell Res.

[48] Li Y, Zhang X, Zheng Q, Zhang Y, Ma Y, Zhu C, Yang L, Peng X, Wang Q, Wang B (2020). YAP1 inhibition in HUVECs is associated with released exosomes and increased hepatocarcinoma invasion and metastasis. Mol Ther Nucleic Acids.

[49] Xu ZH, Miao ZW, Jiang QZ, Gan DX, Wei XG, Xue XZ, Li JQ, Zheng F, Qin XX, Fang WG (2019). Brain microvascular endothelial cell exosome-mediated S100A16 up-regulation confers small-cell lung cancer cell survival in brain. FASEB J.

[50] Sharghi-Namini S, Tan E, Ong LL, Ge R, Asada HH (2014). Dll4-containing exosomes induce capillary sprout retraction in a 3D microenvironment. Sci Rep.

[51] Ju R, Zhuang ZW, Zhang J, Lanahan AA, Kyriakides T, Sessa WC, Simons M (2014). Angiopoietin-2 secretion by endothelial cell exosomes: Regulation by the phosphatidylinositol 3-kinase (PI3K)/Akt/endothelial nitric oxide synthase (eNOS) and syndecan-4/syntenin pathways. J Biol Chem.

[52] Muturi HT, Dreesen JD, Nilewski E, Jastrow H, Giebel B, Ergun S, Singer BB (2013). Tumor and endothelial cell-derived microvesicles carry distinct CEACAMs and influence T-cell behavior. PLoS One.

[53] Serban KA, Rezania S, Petrusca DN, Poirier C, Cao D, Justice MJ, Patel M, Tsvetkova I, Kamocki K, Mikosz A (2016). Structural and functional characterization of endothelial microparticles released by cigarette smoke. Sci Rep.

[54] Ma X, Wang J, Li J, Ma C, Chen S, Lei W, Yang Y, Liu S, Bihl J, Chen C (2018). Loading MiR-210 in endothelial progenitor cells derived exosomes boosts their beneficial effects on hypoxia/reoxygeneation-injured human endothelial cells via protecting mitochondrial function. Cell Physiol Biochem.

[55] Yue Y, Garikipati VNS, Verma SK, Goukassian DA, Kishore R (2017). Interleukin-10 deficiency impairs reparative properties of bone marrow-derived endothelial progenitor cell exosomes. Tissue Eng Part A.

[56] van Balkom BW, de Jong OG, Smits M, Brummelman J, den Ouden K, de Bree PM, van Eijndhoven MA, Pegtel DM, Stoorvogel W, Wurdinger T, Verhaar MC (2013). Endothelial cells require miR-214 to secrete exosomes that suppress senescence and induce angiogenesis in human and mouse endothelial cells. Blood.

[57] de Jong OG, Verhaar MC, Chen Y, Vader P, Gremmels H, Posthuma G, Schiffelers RM, Gucek M, van Balkom BW (2012). Cellular stress conditions are reflected in the protein and RNA content of endothelial cell-derived exosomes. J Extracell Vesicles.

[58] Zhan R, Leng X, Liu X, Wang X, Gong J, Yan L, Wang L, Wang Y, Wang X, Qian LJ (2009). Heat shock protein 70 is secreted from endothelial cells by a non-classical pathway involving exosomes. Biochem Biophys Res Commun.

[59] Liu W, Zhang H, Mai J, Chen Z, Huang T, Wang S, Chen Y, Wang J (2018). Distinct anti-fibrotic effects of exosomes derived from endothelial colony-forming cells cultured under normoxia and hypoxia. Med Sci Monit.

[60] Halkein J, Tabruyn SP, Ricke-Hoch M, Haghikia A, Nguyen NQ, Scherr M, Castermans K, Malvaux L, Lambert V, Thiry M (2013). MicroRNA-146a is a therapeutic target and biomarker for peripartum cardiomyopathy. J Clin Invest.

[61] Gollmann-Tepekoylu C, Polzl L, Graber M, Hirsch J, Nagele F, Lobenwein D, Hess MW, Blumer MJ, Kirchmair E, Zipperle J (2020). miR-19a-3p containing exosomes improve function of ischaemic myocardium upon shock wave therapy. Cardiovasc Res.

[62] Hergenreider E, Heydt S, Treguer K, Boettger T, Horrevoets AJ, Zeiher AM, Scheffer MP, Frangakis AS, Yin X, Mayr M (2012). Atheroprotective communication between endothelial cells and smooth muscle cells through miRNAs. Nat Cell Biol.

[63] Li S, Zhan JK, Wang YJ, Lin X, Zhong JY, Wang Y, Tan P, He JY, Cui XJ, Chen YY (2019). Exosomes from hyperglycemia-stimulated vascular endothelial cells contain versican that regulate calcification/senescence in vascular smooth muscle cells. Cell Biosci.

[64] Chen L, Hu L, Li Q, Ma J, Li H (2019). Exosome-encapsulated miR-505 from ox-LDL-treated vascular endothelial cells aggravates atherosclerosis by inducing NET formation. Acta Biochim Biophys Sin (Shanghai).

[65] He S, Wu C, Xiao J, Li D, Sun Z, Li M (2018). Endothelial extracellular vesicles modulate the macrophage phenotype: Potential implications in atherosclerosis. Scand J Immunol.

[66] Wu XM, Gao YB, Xu LP, Zou DW, Zhu ZY, Wang XL, Yao WJ, Luo LT, Tong Y, Tian NX (2017). Tongxinluo inhibits renal fibrosis in diabetic nephropathy: Involvement of the suppression of intercellular transfer of TGF-[Formula: see text]1-containing exosomes from GECs to GMCs. Am J Chin Med.

[67] Walker JD, Maier CL, Pober JS (2009). Cytomegalovirus-infected human endothelial cells can stimulate allogeneic CD4+ memory T cells by releasing antigenic exosomes. J Immunol.

[68] Barros FM, Carneiro F, Machado JC, Melo SA (2018). Exosomes and immune response in cancer: friends or foes?. Front Immunol.

[69] Carman CV, Martinelli R (2015). T lymphocyte-endothelial interactions: emerging understanding of trafficking and antigen-specific immunity. Front Immunol.

[70] Gavlovsky PJ, Tonnerre P, Guitton C, Charreau B (2016). Expression of MHC class I-related molecules MICA, HLA-E and EPCR shape endothelial cells with unique functions in innate and adaptive immunity. Hum Immunol.

[71] Hosseinkhani B, Kuypers S, van den Akker NMS, Molin DGM, Michiels L (2018). Extracellular vesicles work as a functional inflammatory mediator between vascular endothelial cells and immune cells. Front Immunol.

[72] Wang K, Jiang Z, Webster KA, Chen J, Hu H, Zhou Y, Zhao J, Wang L, Wang Y, Zhong Z (2017). Enhanced cardioprotection by human endometrium mesenchymal stem cells driven by exosomal microRNA-21. Stem Cells Transl Med.

[73] Njock MS, Cheng HS, Dang LT, Nazari-Jahantigh M, Lau AC, Boudreau E, Roufaiel M, Cybulsky MI, Schober A, Fish JE (2015). Endothelial cells suppress monocyte activation through secretion of extracellular vesicles containing antiinflammatory microRNAs. Blood.

[74] Li Q, Nong A, Huang Z, Xu Y, He K, Jia Y, Huang Y (2021). Exosomes containing miR-122-5p secreted by LPS-induced neutrophils regulate the apoptosis and permeability of brain microvascular endothelial cells by targeting OCLN. Am J Transl Res.

[75] Hardy MP, Audemard E, Migneault F, Feghaly A, Brochu S, Gendron P, Boilard E, Major F, Dieude M, Hebert MJ, Perreault C (2019). Apoptotic endothelial cells release small extracellular vesicles loaded with immunostimulatory viral-like RNAs. Sci Rep.

[76] Zhao K, Chen BJ, Chen ZG, Zhang YJ, Xu D, Liu Q (2015). Effect of miR-503 Down-regulation on growth and invasion of esophagus carcinoma and related immune function. Med Sci Monit.

[77] Hou ZH, Xu XW, Fu XY, Zhou LD, Liu SP, Tan DM (2020). Long non-coding RNA MALAT1 promotes angiogenesis and immunosuppressive properties of HCC cells by sponging miR-140. Am J Physiol Cell Physiol.

[78] Schmittnaegel M, Rigamonti N, Kadioglu E, Cassara A, Wyser Rmili C, Kiialainen A, Kienast Y, Mueller HJ, Ooi CH, Laoui D, De Palma M (2017). Dual angiopoietin-2 and VEGFA inhibition elicits antitumor immunity that is enhanced by PD-1 checkpoint blockade. Sci Transl Med.

[79] Zhuang H, Chen X, Dong F, Zhang Z, Zhou Z, Ma Z, Huang S, Chen B, Zhang C, Hou B (2021). Prognostic values and immune suppression of the S100A family in pancreatic cancer. J Cell Mol Med.

[80] Zhang Y, Yang X, Zhu XL, Bai H, Wang ZZ, Zhang JJ, Hao CY, Duan HB (2021). S100A gene family: immune-related prognostic biomarkers and therapeutic targets for low-grade glioma. Aging (Albany NY).

[81] Haney MJ, Klyachko NL, Zhao Y, Gupta R, Plotnikova EG, He Z, Patel T, Piroyan A, Sokolsky M, Kabanov AV, Batrakova EV (2015). Exosomes as drug delivery vehicles for Parkinson's disease therapy. J Control Release.

[82] Tian T, Zhang HX, He CP, Fan S, Zhu YL, Qi C, Huang NP, Xiao ZD, Lu ZH, Tannous BA, Gao J (2018). Surface functionalized exosomes as targeted drug delivery vehicles for cerebral ischemia therapy. Biomaterials.

[83] Nakase I, Futaki S (2015). Combined treatment with a pH-sensitive fusogenic peptide and cationic lipids achieves enhanced cytosolic delivery of exosomes. Sci Rep.

[84] Kalimuthu S, Gangadaran P, Rajendran RL, Zhu L, Oh JM, Lee HW, Gopal A, Baek SH, Jeong SY, Lee SW (2018). A new approach for loading anticancer drugs into mesenchymal stem cell-derived exosome mimetics for cancer therapy. Front Pharmacol.

[85] Lu M, Xing H, Xun Z, Yang T, Ding P, Cai C, Wang D, Zhao X (2018). Exosome-based small RNA delivery: progress and prospects. Asian J Pharm Sci.

[86] Wolburg H, Lippoldt A (2002). Tight junctions of the blood-brain barrier: development, composition and regulation. Vascul Pharmacol.

[87] Aqil F, Munagala R, Jeyabalan J, Agrawal AK, Kyakulaga AH, Wilcher SA, Gupta RC (2019). Milk exosomes - natural nanoparticles for siRNA delivery. Cancer Lett.

[88] Yang T, Fogarty B, LaForge B, Aziz S, Pham T, Lai L, Bai S (2017). Delivery of small interfering RNA to inhibit vascular endothelial growth factor in Zebrafish using natural brain endothelia cell-secreted exosome nanovesicles for the treatment of brain cancer. AAPS J.

[89] Catalano M, O'Driscoll L (2020). Inhibiting extracellular vesicles formation and release: a review of EV inhibitors. J Extracell Vesicles.

[90] Menck K, Sonmezer C, Worst TS, Schulz M, Dihazi GH, Streit F, Erdmann G, Kling S, Boutros M, Binder C, Gross JC (2017). Neutral sphingomyelinases control extracellular vesicles budding from the plasma membrane. J Extracell Vesicles.

[91] Zhao L, Luo H, Li X, Li T, He J, Qi Q, Liu Y, Yu Z (2017). Exosomes derived from human pulmonary artery endothelial cells shift the balance between proliferation and apoptosis of smooth muscle cells. Cardiology.

[92] Bobrie A, Krumeich S, Reyal F, Recchi C, Moita LF, Seabra MC, Ostrowski M, Thery C (2012). Rab27a supports exosome-dependent and -independent mechanisms that modify the tumor microenvironment and can promote tumor progression. Cancer Res.

[93] Huang Z, Feng Y (2017). Exosomes derived from hypoxic colorectal cancer cells promote angiogenesis through Wnt4-induced beta-catenin signaling in endothelial cells. Oncol Res.

